# DNA Damage Repair: Predictor of Platinum Efficacy in Ovarian Cancer?

**DOI:** 10.3390/biomedicines10010082

**Published:** 2021-12-31

**Authors:** Dimitra T. Stefanou, Vassilis L. Souliotis, Roubini Zakopoulou, Michalis Liontos, Aristotelis Bamias

**Affiliations:** 1 First Department of Medicine, Laiko General Hospital, School of Medicine, National and Kapodistrian University of Athens, 11527 Athens, Greece; dimitroulastef@hotmail.com; 2 Institute of Chemical Biology, National Hellenic Research Foundation, 11635 Athens, Greece; vls@eie.gr; 3 2nd Propaedeutic Department of Internal Medicine, Attikon University Hospital, National and Kapodistrian University of Athens, 12462 Athens, Greece; Rzakopoul@gmail.com; 4 Department of Clinical Therapeutics, Alexandra General Hospital, School of Medicine, National and Kapodistrian University of Athens, 11528 Athens, Greece; mliontos@gmail.com

**Keywords:** ovarian cancer, DNA repair, platinum drugs, effective biomarkers, therapeutic targets

## Abstract

Ovarian cancer (OC) is the seventh most common type of cancer in women worldwide. Treatment for OC usually involves a combination of surgery and chemotherapy with carboplatin and paclitaxel. Platinum-based agents exert their cytotoxic action through development of DNA damage, including the formation of intra- and inter-strand cross-links, as well as single-nucleotide damage of guanine. Although these agents are highly efficient, intrinsic and acquired resistance during treatment are relatively common and remain a major challenge for platinum-based therapy. There is strong evidence to show that the functionality of various DNA repair pathways significantly impacts tumor response to treatment. Various DNA repair molecular components were found deregulated in ovarian cancer, including molecules involved in homologous recombination repair (HRR), nucleotide excision repair (NER), mismatch repair (MMR), non-homologous end-joining (NHEJ), and base excision repair (BER), which can be possibly exploited as novel therapeutic targets and sensitive/effective biomarkers. This review attempts to summarize published data on this subject and thus help in the design of new mechanistic studies to better understand the involvement of the DNA repair in the platinum drugs resistance, as well as to suggest new therapeutic perspectives and potential targets.

## 1. Ovarian Cancer

Ovarian cancer (OC) represents the seventh most common type of cancer for women worldwide, with a frequency of 4% of all new cancer cases in females annually. It is associated with the highest fatality rate among gynecological cancers, mainly because of delayed diagnosis. Indeed, the majority of early-stage ovarian cancers is asymptomatic and cannot be easily diagnosed [1,2].

According to the 2020 World Health Organization (WHO) classification, at least five main types of ovarian carcinomas are identified based on morphology: high-grade serous carcinoma (HGSC, the most prevalent subtype of ovarian cancer; 70%), endometrioid carcinoma (EC, 10%), clear cell carcinoma (CCC, 6–10%), low-grade serous carcinoma (LGSC, 5%), and mucinous carcinoma (MC, 3–4%) [3]. These histological subtypes display distinct molecular characteristics both at the genomic and transcriptomic level. For example, the most frequent molecular defect in HGSC at the genomic level is the TP53 mutation [4]. Interestingly, about half of HGSC have identifiable germline, somatic, or epigenetic defects in the homologous recombination DNA repair (HRR) pathway, with most of these defects being germline or somatic BRCA1 (breast cancer type 1 susceptibility protein) or BRCA2 (breast cancer type 2 susceptibility protein) mutations, which together account for approximately 20% of cases [5,6,7]. Mutations deregulating the PI3K pathway are also common in the endometrioid OC; around 20% of cases harbor PTEN tumor suppressor gene mutations and around 30% display activating PIK3CA mutations [8]. Endometrioid OC also commonly displays activated Wnt signaling, with about half showing CTNNB1 mutation. Such as endometrioid OC, clear cell carcinomas harbor defects in PTEN (~10% of cases), PIK3CA (~50% of cases), and ARID1A (~50% of cases), consistent with the shared molecular pathogenesis and developmental origins of these carcinomas [8]. Moreover, KRAS mutation and HER2 gene amplification are known common events in mucinous OC, with 50% and 20% of cases displaying these defects, respectively [9]. All the above characteristics are connected not only with the prognosis, but also with the choice of therapeutic interventions.

Ovarian cancer is primarily staged using the FIGO (International Federation of Gynecology and Obstetrics) staging system [10]. The system is based mainly on local, regional, and distant cancer’s spread and is closely related to prognosis. Thus, stage I OC patients have a 5-year survival rate of 90%, stage II of 70%, and stage ΙΙΙ of ~39%, whereas stage VI patients show a rate of 17%. Unfortunately, most patients are diagnosed with stage III or IV disease, which underlines the significant need for further progress in the management of this disease.

## 2. Treatment of Advanced Ovarian Cancer

The mainstay of front-line treatment of advanced OC has not changed in the last decade. Patients are submitted to cytoreductive surgery aiming to achieve minimal or no residual disease and to systemic chemotherapy with the combination of carboplatin and paclitaxel, before or after cytoreductive surgery. Although the response rate for this first-line approach is 70–80%, with more than 50% achieving complete remission after surgery and chemotherapy, the majority of patients with advanced ovarian cancer will subsequently relapse or progress and require further intervention, which again may include the combination of chemotherapy and surgery, although the role of the former is increasingly more critical as the disease continues to relapse. Platinum-based chemotherapy can be successfully used in relapses of the disease provided that the time period from the end of prior platinum therapy is more than six months. It is, therefore, evident that platinum is essential for patients in many phases of their disease and resistance to this agent signals a significant worsening of their prognosis [11].

**Figure 1:**
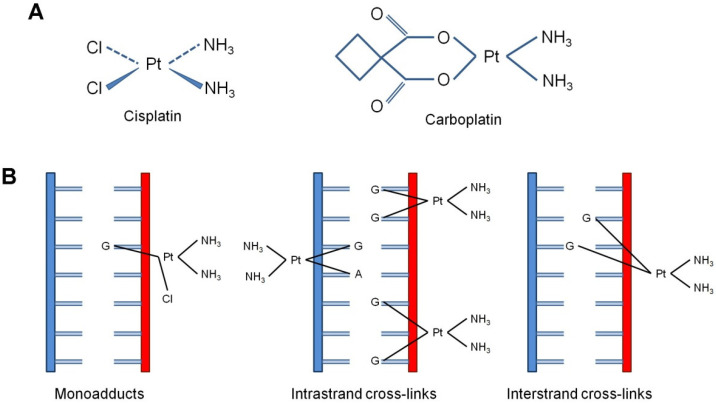
Cisplatin-induced DNA adducts. (**A**) Structure formulas of cisplatin and carboplatin. (**B**) The type of DNA adducts formed by cisplatin: single-nucleotide damage of guanine (monoadducts), intra-strand cross-links [Pt-d(GpG)], 1,2-intra-strand crosslinks, 65%; Pt-d(ApG), 1,2-intra-strand cross-links, 25%; Pt-d(GpNgG), 1,3-intra-strand cross-links, 5–10%] and inter-strand cross-links (1.5%).

The mechanism of action of both cisplatin and carboplatin includes their interaction with DNA and the formation of monoadducts, mostly covalently interacting with N7-position of guanine. Afterwards, this monoadduct evolves, through a second covalent binding, to a DNA cross-link, which can be on the same DNA strand (intra-strand) or on the opposite strand (inter-strand). The kinetics of monoadduct and monoadduct to cross-links formation are the most important differences between cisplatin and carboplatin, owing to various aquation rates and steric hindrance (Figure 1).

In response to these genotoxic insults, the DNA damage response (DDR) network orchestrates DNA damage checkpoint activation and facilitates the removal of DNA lesions [12]. Indeed, following detection of DNA lesions, there is an induction of a signal transduction cascade, including molecules that activate genome-protection pathways, such as DNA repair, cell cycle control, apoptosis, transcription, and chromatin remodeling. As DDR is a signal transduction pathway that determines the cell’s ability to repair DNA damage or to undergo apoptosis, its role has been implicated in the disease process and the successful outcome of chemotherapy [13]. Notably, another important type of DNA damage induced by platinum-based drugs is covalent DNA-protein crosslinks. This includes the crosslinking of histones but also of potentially any protein in the vicinity of DNA [14]. DNA-protein crosslinks are particularly toxic DNA lesions, as they impede fundamental DNA processes. More importantly, there is evidence indicating a positive correlation between the clinical efficacy of platinum-based compounds and the extent to which they induce DNA-protein crosslinks [15,16].

**Figure 2:**
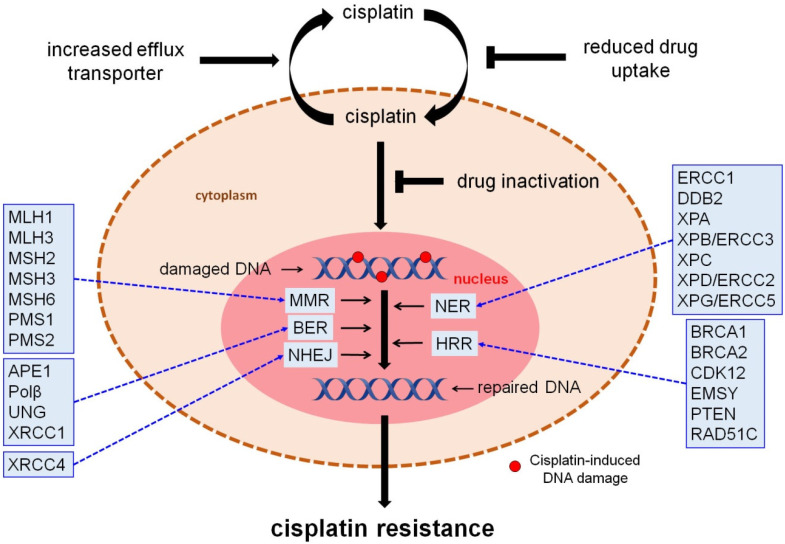
Molecular mechanisms of cisplatin resistance. Cells block cisplatin from damaging DNA by decreasing drug uptake, increasing drug efflux, and augmenting drug detoxification by binding to glutathione or metalloproteins. Following DNA damage induction, cells remove the lesions using critical DNA repair mechanisms. Molecular components that were found deregulated in OC, including MLH1, MLH3, MSH2, MSH3, MSH6, PMS1, PMS2 (MMR), APE1, Polβ, UNG, XRCC1 (BER), XRCC4 (NHEJ), ERCC1, DDB2, XPA, XPB/ERCC3, XPC, XPD/ERCC2, XPG/ERCC5 (NER) and BRCA1, BRCA2, CDK12, EMSY, PTEN, and RAD51C (HRR) can be possibly exploited as novel therapeutic targets and sensitive/effective biomarkers.

Resistance to cisplatin has been associated with multiple mechanisms, including tumor heterogeneity, reduced drug uptake, alterations in pro-survival and pro-apoptotic pathways, modification of a drug target, inactivation of the drug, and upregulation of DNA repair pathways. In fact, there is strong evidence to show that the functionality of various DNA repair pathways significantly impacts tumor response to cisplatin treatment [17,18]. Guided by this notion, this review attempts to summarize aspects of published data on this subject and thus help in the design of new mechanistic studies to better understand the involvement of the DDR network in the platinum-based drugs resistance, as well as to suggest new therapeutic perspectives and potential targets (Figure 2).

## 3. DNA Repair Responses to Cisplatin-Induced DNA Damage

### 3.1. Homologous Recombination Repair (HRR)

HRR is an error-free DNA repair mechanism, which operates during the S and G2 phases of the cell cycle so that it can find a large area of homology on a sister chromatid to use as a template for resynthesizing damaged or lost bases [19]. HRR begins with nucleolytic resection of DNA ends, mediated by the combined action of the MRN (Mre11/Rad50/Nbs1) complex, CtIP, and BRCA1, which yields 3′ single-stranded DNA tails that are stabilized by the replication protein A (RPA). Then, BRCA2 catalyzes the displacement of RPA protein and the formation of a RAD51 nucleoprotein filament, which promotes homology search and catalyzes an exchange strand between the broken duplex and an intact sister chromatid. The 3′-end of the invading strand is extended by DNA synthesis using the sister chromatid as a template, and intact duplexes are eventually restored using a resolution mechanism [19,20]. As mentioned above, up to 50% of high-grade serous ovarian cancer displays homologous recombination deficiency (HRD). The most frequently noticed and well-studied mutations are observed in the BRCA1 and BRCA2 genes and may be germline or somatic mutations [21] (Table 1). Of note, defective HR in OC may also occur via alterations in genes that are indirectly modulating the HR pathway and thus cause HR deficiency [22]. For example, a focal deletion region at 10q23.31 that includes only the phosphatase and tensin homolog (PTEN) gene has been found in approximately 7% of high-grade serous OCs [23]. Moreover, several studies have reported both overexpression and amplification of the BRCA2-Interacting Transcriptional Repressor (EMSY) gene as another mechanism of HR deficiency in as high as 17% of high-grade sporadic OC [24]. Cyclin-dependent kinase 12 (CDK12) is also one of the only nine significantly mutated genes in ovarian cancer (3% of cases in the TCGA dataset) and is known to promote the transcription of several HR pathway genes, including BRCA1 [25]. In addition, individuals with a RAD51 Homolog C (RAD51C) mutation have an increased risk to develop ovarian cancer [26].

Nowadays, a major number of assays are under investigation in order to classify tumors as HR-proficient or HR-deficient. In general, there are two main methods to classify tumors: methods that determine genomic alterations and those that determine the function of proteins. The first ones indicated (BROCA, Myriad, Foundation Medicine, HRD detect) adopt techniques such as NGS or whole genome sequencing. These assays quantitate genomic instability in a tumor genome based on three independent measures of genomic instability, and they can be summarized in the loss of telomeric allelic imbalance (TAI), loss of heterozygosity (LOH), and large-scale transition (LST). The above-mentioned parameters have been estimated not only as a whole but also separately, and it is worth stating that the combination of the approaches offers the most valid results. Moreover, it can be inferred that it is a convenient assay owing to the fact that it uses blood tests [27,28]. In addition, the tissue-based assay foundation focus on CDx examines both germline and somatic mutations in a tumor, while the Myriad Genetics BRCA analysis CDx platform identifies only germline mutations in blood. Both of them are FDA approved so as to determine the subgroup of OC patients, who may benefit from treatment with poly (ADP-ribose) polymerase inhibitors (PARPi). Other efforts to validate assays have been performed in order to indicate the functionality of proteins related to HR. One of them detects RAD51 foci formation, a single downstream event of HR activation. On the whole, the above assays show some limitations such as the difficult processing of formalin-fixed paraffin-embedded (FFPE) tissue specimens, the formation of large amounts of artifacts, the difficulty of post-treatment biopsies in clinical practice, the unidentified timing of RAD51 foci formation, and false negative results. [29,30]

Accumulated data have shown that the HRD approach is a positive predictor of response to platinum-based drugs [31]. The response is relied not only on progression free survival (PFS) or treatment free interval (TFI), but also on overall survival (OS), since the method used to estimate HRD did not focus only on the detection of BRCA and other genes mutations but gave emphasis on other alterations having already been mentioned [32,33,34]. It is worth noting that a few studies prove that a mutation on BRCA2 and not on BRCA1 is related to the response to platinum. It is possibly attributed to the fact that the two genes have not only complementary but also distinct functions. However, this fact concerns a limited number of patients and previous studies, and there is a necessity of further research to confirm the findings [21]. Additionally, the polyclonality of the tumor or the existence of more than one responsible mechanism is of great importance in clinical practice, as has already been validated by SCOTROC4 clinical trial. Through this trial, it has also been confirmed that OC patients with BRCA mutations have HR deficiency and are characterized by increased platinum sensitivity.According to a study of exploratory analysis, a decrease of threshold of HDR score in lower levels than 33 appears to be a result of platinum sensitivity. Of note, a previous analysis of plasma has shown that platinum dose-intensification may benefit the drug-sensitive subpopulation of OC patients [31]. It is true that there are negatives results, too. That means that the existence of mutations in BRCA genes or “BRCAness” condition is not connected with the response to platinum [32,33].

Furthermore, there are important findings related to drug-induced restoration of HR due to the selective pressure of using platinum as treatment. This restoration occurs by multiple different mechanisms, such as reversion mutations or intragenic deletions in BRCA1 and 2 mutated genes or the loss of BRCA1 promoter methylation. In essence, through the above mechanisms, the protein frame is restored resulting in the formation of functional protein and the re-acquiring of HR adequacy. These alterations can be detected by examining cell-free DNA, progression biopsies, or germline DNA samples. As for the last method followed, practical obstacles are mentioned, since re-biopsy in OC is not a clinical practice [35,36]. Another mechanism of HR restoration is HSP90-mediated stabilization of a BRCA C-terminal (BRCT) domain of BRCA mutant BRCA1 protein under platinum treatment. The stabilized mutant BRCA1 protein confers cisplatin resistance due to interaction with PALB2-BRCA2-RAD51, which is essential for RAD51 focus formation.Interestingly, it has been demonstrated that mutation RAD51C/RAD51D in post progression tumor biopsy samples collected from patients in ARIEL2 Part 1, a phase II study of the PARPi rucaparib as treatment for platinum-sensitive, relapsed ovarian carcinoma is a mechanism of restoration of HR and acquired PARPi resistance [35,37,38,39].

### 3.2. Nucleotide Excision Repair (NER)

NER is a fundamental DNA repair mechanism involved in the removal of bulky, helix-distorting lesions from DNA [40]. In literature, NER pathway was first mentioned in 1980 by Haisson and his partners. Since then, research has been conducted so as to determine the sequence of the molecular events and the significance of each component. NER is an essential DNA repair mechanism involved in the removal of bulky, helix-distorting lesions from DNA. DNA adducts that are repaired by NER involve cyclobutane pyrimidine dimers (CPDs) and 6-4 photoproducts (6-4 PPs) produced by UV radiation, DNA lesions generated by reactive oxygen species (ROS), or endogenous lipid peroxidation products, intra-strand cross-links and adducts produced by genotoxic drugs (melphalan, cisplatin), or environmental carcinogens (benzo[a]pyrene). Two sub-pathways of NER could be mentioned, termed GGR (global genome repair) and TCR (transcription-coupled repair), where almost 30 proteins are involved in each one. As for the first step, the recognition of DNA damage differs between the two sub-pathways. In GGR, the formation of a bulky DNA adduct induces an increase in helix distortion, which facilitates the recruitment of the damage recognition factor XPC/RAD23/CETN2 and UV-DDB. Nevertheless, damage recognition in TCR is initiated when an elongating RNA polymerase II (RNAPII) is arrested upon encountering a site of DNA damage. In both GGR and TCR sub-pathways, the damaged duplex DNA is opened around the lesion by TFIIH, a multi-subunit helicase complex that includes XPB, p62, p52, p44, p34, p8, XPD, and the CDK-activating kinase (CAK). Next, the structure-specific endonucleases XPG and XPF/ERCC1 excise DNA 3′ and 5′ to the lesion, to eliminate a 25–30 nt-long oligonucleotide, including the DNA damage. Finally, new DNA is synthetized by the DNA polymerases delta (Pol δ), kappa (Pol κ), and epsilon (Pol ε), using the intact strand as template, and terminated by the XRCC1/ligase 3 [40].

Taking into consideration the vital role of NER in the repair of platinum drug-induced DNA damage, it has been attempted to associate NER deficiency with patients’ sensitivity to platinum [41]. Interestingly, about 8% of OC patients showing alterations in NER-associated genes are characterized by augmented OS and PFS. However, during the last decade, there is no medical interest due to conflicting results and the fact that more and more studies agree that the time of evaluation of the deficiency affects the result. In the majority of studies, deficiency was evaluated before administering platinum. According to further studies, it has been proven that low NER protein expression after platinum treatment is associated with platinum resistance [42]. Most studies have quantified not only protein levels of NER components but also mRNA or gene polymorphisms. Other studies use cell lines deficient in NER proteins [43].

As for the GGR sub-pathway of NER, a previous study assessed the association of 22 single-nucleotide polymorphisms (SNPs) of the GGR repair gene xeroderma pigmentosum, complementation group C (XPC) with PFS in patients with advanced ovarian cancer who underwent primary cytoreductive surgery followed by platinum-based chemotherapy (Table 1). Three SNPs were associated significantly with prolonged PFS in that cohort, suggesting that SNPs in this gene may represent novel markers of ovarian cancer response to platinum-based chemotherapy [44]. Moreover, the results of another study showed that the XPC protein is involved in the cisplatin DNA damage-mediated signaling process, suggesting that this protein plays an important role in eliminating damaged cells and in preventing cancer occurrence and that XPC defects can lead to a high risk of cancer occurrence [45]. In addition, other reports have confirmed that the damage-specific DNA binding protein 2 (DDB2) gene, a molecular GGR/NER component that also plays an important role in apoptosis, participates in the sensitivity of platinum-based drugs in ovarian carcinoma cells [46,47].

On the other hand, deficiency of the TCR-NER sub-pathway correlates with platinum resistance. Below, it is attempted to describe data concerning TCR disfunction related to cisplatin sensitivity. In this way, predictive markers of response in cisplatin treatment could be found [48].

The xeroderma pigmentosum complementation group D (XPD) gene, also known as ERCC2, plays important roles in the nucleotide NER pathway. It encodes an evolutionarily conserved helicase that participates in DNA unwinding and the recognition of bulky adducts and thymidine dimers. Previous studies have shown that the XPD gene polymorphism Lys751Gln may be associated with an increased risk of ovarian carcinoma [49], as well as increased PFS and OS following platinum treatment [50,51]. Moreover, the xeroderma pigmentosum, complementation group B (XPB/ERCC3) gene encodes an ATP-dependent DNA helicase that functions in TCR/NER; the encoded protein is a subunit of basal transcription factor 2 (TFIIH) and, therefore, also functions in class II transcription. A previous study has shown that the mRNA levels of the XPB gene were higher in clear cell tumors as opposed to other types of epithelial ovarian cancer. This is consistent with the long-standing observation that clear cell tumors are more likely to show de novo drug resistance against DNA damaging agents in the clinic [52]. On the other hand, other studies investigating XPB and XPD, both at the mRNA level and gene polymorphisms, did not find any correlation with cisplatin resistance in ovarian carcinoma [18].

The xeroderma pigmentosum, complementation group A (XPA) gene seems to be involved during UV damage recognition in both GGR/NER and TCR/NER. XPA is a first-order clock-controlled protein, and as a consequence, the rate of NER exhibits high-amplitude circadian rhythmicity [53]. Because NER plays a crucial mechanism for removing the predominant DNA adducts induced by the anticancer drug cisplatin, the repair rates of these adducts exhibit circadian rhythmicity in brain, liver, kidney, skin, and all other tissues tested [54]. Thus, in tumors arising in tissues with circadian rhythmicity, provided the tumor maintains rhythmicity in phase with the normal tissue, administration of cisplatin when excision repair is in the descending phase is expected to improve the therapeutic index by administering a less toxic dose. Furthermore, during the sequencing of events and the activation of NER, XPA protein interacts with ERCC1 protein, and it has been indicated that the quantification of XPA level with ERCC1 in mRNA and protein level is the most reliable indicator of response to platinum.

The excision repair cross complementing-group 1 (ERCC1) component is a mammalian endonuclease that incises the damaged strand of DNA during NER and inter-strand cross-link repair. Current studies have shown contradictive results concerning ERCC1 as a predictive factor of response to platinum treatment. That is the reason why measurement of ERCC1 is not in clinical practice despite having been studied since 1990. All the assessments have taken place in mRNA level, protein level. or gene expression level in order to detect particular SNPs. As for the conflicting results, they may be due to lack of validated antibodies working in immune-histochemistry or evaluation of all the isoforms of ERCC1 [55,56]. In an effort to overcome the above limitations, new antibodies that recognize isoform ERCC1 related to XPF molecule have been discovered. Moreover, new methods are used to recognize the active ERCC1 isoform XPF complex, which implies NER proficiency [57]. Relative method is PLA-proximity ligation assay and the first findings connecting ERCC1 functional proficiency with cisplatin sensitivity in OC have already been published. Interestingly, a previous study reported that the expression of ERCC1 in circulating tumor cells may be used as an independent predictive biomarker for platinum-resistance and poor prognosis of ovarian cancer [58].

The xeroderma pigmentosum, complementation group G (XPG/ERCC5) component of NER, as a complex with ERCC1, is a structure-specific endonuclease that makes the 3’ incision in DNA excision repair following UV-induced damage. Interestingly, previous studies have shown that low XPG expression is associated with good cisplatin response in ovarian patients [59].

Taken together, NER recognizes DNA crosslinks caused by platinum and converts them into DNA double strand breaks, which are repaired through the activation of HR. It has been proven that either NER or HR deficiency leads to sensitivity to platinum on account of the accumulation of irreversible DNA damages, and as a consequence, the process of apoptosis is activated. However, in case both mechanisms do not work properly, such as a mutation in NER genes in BRCA mutation background, the cell is not sensitive to platinum. Particularly, due to NER deficiency, double strand breaks will not be formed, and the cell will activate other mechanisms and pathways in order to repair DNA damage.

### 3.3. Mismatch Repair (MMR)

MMR system is an important contributor to replication fidelity, removing base substitution and insertion/deletion mismatches that arise because of replication errors escaping the proofreading function of DNA polymerases. It is consisted of seven molecular components (MutS homolog 2 (MSH2), MutS homolog 3 (MSH3), MutS homolog 6 (MSH6), MutL homolog 1 (MLH1), MutL homolog 3 (MLH3), post meiotic segregation increased 1 (PMS1), and post meiotic segregation increased 2 (PMS2)), whose alterations are the second most common cause of hereditary OC, following BRCA1 and BRCA2 mutations (Table 1) [60]. Previous reports have shown that the incidence of germline MMR gene mutations in OC is only 2%. However, other mechanisms of gene inactivation (such as promoter hypermethylation) leading to the loss of expression of one of the seven main MMR genes also occur in up to 29% of cases [61]. The recognition of DNA lesions is accomplished by the complex MUTSα, a heterodimer of the DNA mismatch repair proteins MSH2 and MSH6. Another heterodimer complex, called MUTSβ (MSH2/MSH3), is able to bind only to insertion/deletion mismatches. Lesion recognition is followed by the recruitment of MutLα (MLH1/PMS2) or MutLβ (MLH1/MLH3), which have endonuclease activity that can incise DNA near the mismatch. The nick is used by the 5′-exonuclease-1 (Exo1) as an entry point to degrade DNA past the mismatch, the resulting single-stranded DNA gap is filled in by the Pol δ and finally sealed with DNA ligase I [62,63].

One of the most important issues is that the clinical characteristics of the subgroup of OC patients related to MMR genes alterations have not been identified yet. Nevertheless, according to the majority of the findings, MMR deficiency is associated with sensitivity to platinum-based drugs. For example, MSH2 can interact with ATR and recruit it to the sites of DNA damage, further activating a series of apoptosis proteins and resulting in apoptosis of the cells. Therefore, inactivation of this important MMR component plays a crucial role in platinum resistance of ovarian cancer [64]. In line with these data, another study suggested that the expression profile of hMSH2 could be a potential prognostic biomarker in epithelial ovarian cancer [65]. In addition, studies in ovarian cancer have reported a frequency of MLH1 promoter hypermethylation that ranges between 10% and 50%, with the higher estimates reported in microsatellite instability high (MSI-H) tumors [66,67].

### 3.4. Non-Homologous End-Joining (NHEJ)

NHEJ is an important pathway in eukaryotic cells responsible for the repair of DNA double-strand breaks (DSBs) throughout the cell cycle, including during S and G2 phases [68]. It is essential to mention that in the absence of BRCA1, DNA double strand breaks may be repaired by NHEJ pathway and 40% of OC patients display defective NHEJ. NHEJ starts with the recognition of DNA ends by Ku70/80 and is followed by the recruitment and activation of the DNA-dependent protein kinase (DNA-PKcs), and of DNA end-processing enzymes such as Artemis and template-independent polymerases (polymerases λ and μ) that might be necessary for end ligation by the XLF-XRCC4-DNA ligase 4 complex [68]. A critical protein required for NHEJ is the DNA ligase IV (LIGIV) accessory factor, X-ray cross complementing 4 (XRCC4). XRCC4 is believed to stabilize LIGIV, participate in LIGIV activation, and help tether the broken DSB ends together (Table 1). Previous studies have shown that overexpression of the XRCC4 gene was dramatically linked to worse PFS and OS for patients with serous ovarian carcinoma, suggesting the prognostic significance of XRCC4 in serous and endometrioid ovarian carcinomas patients [69].

### 3.5. Base Excision Repair (BER)

BER is a conserved DNA repair pathway that removes damaged DNA bases that do not considerably distort the structure of the DNA helix. It is considered to play an important role in the repair of small base lesions such as alkylations and oxidations [70]. BER consists of two sub-pathways, known as single-nucleotide or short-patch and long-patch; the activation of one or the other is predicated by the cause and type of damage, the type of abasic (AP; apurinic/apyrimidinic) site generated in the first repair step, and the cell cycle phase in progress when the damage occurs. The short-patch pathway quickly repairs single-base damage during the G1 phase of the cell cycle, while the long-patch pathway handles lengthier repair during the S or G2 phases when resynthesis of 2–8 nucleotides surrounding the AP-site is required. Among the enzymes that take part in BER, DNA glycosylases and mono- or bi-functional, are the most important. They recognize and hydrolyze the N-glycosylic bond between the damaged base and the sugar phosphate backbone, creating an AP intermediate site.

Accumulated data have shown that BER plays an important role in mediating the cytotoxicity of ICL-inducing agents (including platinum drugs) and modulates cisplatin cytotoxicity via specific AP endonuclease 1 (APE1), uracil-DNA glycosylase (UNG), and DNA polymerase β (Polβ) functions (Table 1). Indeed, a previous study has shown that the inhibition of human major AP endonuclease 1 and APE1, combined with the knockdown of UNG and Polβ, makes cancer cells more resistant to cisplatin [70]. Interestingly, the authors showed that despite the presence of ICL, UNG excises neighboring uracil residues to generate AP sites, which are then cleaved by APE1, followed by the Polβ-catalyzed gap-filling DNA repair synthesis. This futile BER adjacent to cisplatin ICL sites initiated by the DNA glycosylase-mediated excision generates persistent DNA strand breaks, which would interfere with the productive repair of ICLs and increase cisplatin cytotoxicity [71]. Of note, previous reports have shown a correlation between the abnormal cytoplasmic level of APE1 and platinum resistance.

Finally, a previous report has shown that the expression of the X-ray repair cross-complementing gene 1 (XRCC1), a critical factor in BER and single strand break repair pathway, has clinicopathological significance and predicts resistance to platinum therapy in ovarian cancer patients [72].

**Table 1:** Critical factors implicated in the repair of cisplatin-induced DNA damage.

DNA Repair Pathway	Symbol	Description	Reference
Homologous recombination repair (HRR)	BRCA1	Breast cancer type 1 susceptibility protein	Pietragalla et al. [6]
	BRCA2	Breast cancer type 2 susceptibility protein	Pietragalla et al. [6]
	CDK12	Cyclin-dependent kinase 12	Joshi et al. [25]
	EMSY	BRCA2-interacting transcriptional repressor EMSY	Hughes-Davies et al. [24]
	PTEN	Phosphatase and tensin homolog	The Cancer Genome Atlas Research Network [23]
	RAD51C	RAD51 homolog C	Hurley et al. [26]
Nucleotide excision repair (NER)	ERCC1	Excision repair cross-complementation, group 1	Chebouti et al. [58]
	DDB2	Damage-specific DNA binding protein 2	Cui et al. [46]
	XPA	Xeroderma pigmentosum, complementation group A	Kang et al. [53]; Sancar et al. [54]
	XPB/ERCC3	Xeroderma pigmentosum, complementation group B	Reed et al. [52]; Damia et al. [18]
	XPC	Xeroderma pigmentosum, complementation group C	Wang et al. [45]; Fleming et al. [44]
	XPD/ERCC2	Xeroderma pigmentosum, complementation group D	Michalska et al. [49]; Kang et al. [50]
	XPG/ERCC5	Xeroderma pigmentosum, complementation group G	Walsh et al. [59]
Mismatch repair (MMR)	MLH1	MutL homolog 1, colon cancer, nonpolyposis type 2	Gras et al. [66]; Kawashima et al. [67]
	MLH3	MutL homolog 3	Zhao et al. [60]
	MSH2	MutS homolog 2, colon cancer, nonpolyposis Type 1	Pabla et al. [64]
	MSH3	MutS homolog 3	Zhao et al. [60]
	MSH6	MutS homolog 6	Zhao et al. [60]
	PMS1	PMS1 post meiotic segregation increased 1	Zhao et al. [60]
	PMS2	PMS2 post meiotic segregation increased 2	Zhao et al. [60]
Non-homologous end-joining (NHEJ)	XRCC4	X-ray repair cross complementing 4	Liu et al. [69]
Base excision repair (BER)	APE1	Apurinic/apyrimidinic endo deoxyribonuclease 1	Kothandapani et al. [70,71]
	Polβ	DNA polymerase beta subunit	Kothandapani et al. [70,71]
	UNG	Uracil-DNA glycosylase	Kothandapani et al. [70,71]
	XRCC1	X-ray repair cross complementing 1	Abdel-Fatah et al. [72]

## 4. New Therapeutic Perspectives in Epithelial Ovarian Cancer

### 4.1. PARP Inhibition in Epithelial Ovarian Cancer

The clinical development of PARPi has significantly altered our approach to OC care. Due to its cytotoxic effects via synthetic lethality, PARPi have been authorized for the treatment of advanced OC in both relapsed and front-line scenarios [73,74]. The US Food and Drug Administration (FDA) and the European Medicines Agency (EMA) have authorized three PARP inhibitors for ovarian cancer: Olaparib, Rucaparib, and Niraparib. All three PARP inhibitors have shown substantial improvements in objective response rate and progression-free survival (PFS) in patients with relapsed platinum-sensitive ovarian cancer and are approved for this setting [75,76,77].

The PARP enzymes are involved in a variety of cellular processes that govern energy consumption as well as gene transcription, cell death, and epigenetic alterations [78]. PARP and BRAC1/2, both of which are essential in DNA double-strand break repair [79], are linked by a synthetic lethal connection. Permanent single-strand DNA breaks (SSBs), which are normally repaired by active base-excision repair pathways, become more common as a result of PARP inhibition, resulting in a buildup of double strand breaks. [73,80,81]. Despite PARPi sharing common mechanisms of action with platinum salts, PARP trapping could signify therapeutic implications for these agents also for patients’ resistance to platinum [82].

Indeed, PARPi have shown efficacy as monotherapy in individuals with platinum-resistant advanced OC, who also have BRCAm. Olaparib had excellent outcomes in platinumresistant EOC in a phase II study involving patients with BRCA mutations and advanced cancer; ORR was 26.2% and stable disease (SD) was reached in 40% of patients [77]. Additionally, it was demonstrated in a pooled analysis of phase I/II studies with Olaparib monotherapy in advanced BRCA mutant cancer that patients with extensively pretreated OC, who were naive to PARPi, had rather persistent responses (RR 31%, median duration of response 7.8 months) [78]. Rucaparib was also licensed by the FDA as monotherapy for BRCA mutant platinum-resistant illness, based on encouraging results from a pooled analysis of phase II studies that demonstrated a 25% response rate [69]. Additionally, Niraparib was examined as monotherapy in recurrent OC in the fourth or subsequent line of treatment in the phase III QUADRA study. ORR was 33% in patients with platinum-resistant disease and 19% in patients with platinum-refractory disease (defined as progression within one month after the last dose of platinum) in the BRCA mutant group, with a median duration of response of 9.2 months [79]. Thus, the FDA authorized Niraparib as monotherapy in platinum-resistant and platinum-refractory illness in October 2019.

Resistance to PARPi is a typical occurrence in advanced OC following a period of effective therapy. Three general mechanisms can result in acquired resistance to PARPi: restoration of HRR as a result of the recovery of BRCA1/2 function or the reversal of DNA end-protection; restoration of replication fork stability; drug target-related effects, such as the upregulation of drug efflux pumps, mainly of the transporter ABCB1, also known as P-glycoprotein, or mutations in PARP and functionally related proteins [83]. BRCA1 or BRCA2 secondary “revertant” mutations, which restore the genes’ open reading frames and adequate HRR function, have been completely confirmed as a resistance mechanism to PARPi, which also results in resistance to platinum-based treatment [84,85]. This has been clearly shown in the SOLO-2 trial [86]. An exploratory analysis of the study evaluated efficacy of post progression chemotherapy [87]. Patients in the Olaparib arm had significantly decreased mPFS with the subsequent chemotherapy, and this difference was most pronounced among patients receiving platinum-base therapy. Therefore, further development of agents that will overcome resistance to PARPi is an unmet medical need and a field of extensive research.

### 4.2. CHK1/2, ATR Inhibitors

Deficiency in DNA repair mechanisms as well as administration of DNA damaging agents, including platinum agents and PARP inhibitors, induce replication stress. In response to replication stress, the DDR pathway is activated, mediated by apical (ATM and ATR) and downstream (CHK1 and CHK2) serine threonine protein kinases, halting cell cycle progression, and initiating DNA repair mechanisms [88]. Thus, targeting DDR kinases has a strong scientific rationale in ovarian carcinomas and could exert synergistic activity with PARP inhibitors. Indeed, the dual CHK1/2 inhibitor prexasertib has shown activity in preclinical models as monotherapy or in combination with Olaparib [89]. In patients with recurrent ovarian cancer, prexasertib also demonstrated a 33% overall response rate [90], and the drug is further evaluated in a BRCA mutant population [91].

ATR inhibition also impairs cell cycle progression and selective ATR inhibitors, including ceralasertib (AZD6738) and berzosertib (M6620/VX-970/VE-822), have shown anti-tumor activity both as single agents or in combination with DNA-damaging chemotherapy, irradiation, and PARP inhibitors [92]. In a phase II trial enrolling patients with platinum resistant ovarian cancer, berzosertib in addition to gemcitabine prolonged mPFS in comparison to gemcitabine alone [93]. Of interest, the benefit was limited to patients that did not harbor genomic replication stress alterations [94]. Currently, several studies with selective ATR inhibitors are ongoing testing combinations with chemotherapy or PARP inhibitors in recurrent ovarian cancer patients [95,96].

### 4.3. Wee1 Inhibitors

WEE1 mediates G2/M transition and induces cell cycle arrest upon DNA damage to allow for DNA repair. Cancer cells selectively rely on G2 arrest to avoid mitotic catastrophe [97]. Under this perspective, WEE1 inhibition has been investigated in several neoplasms in phase I trials [98] showing efficacy in ovarian and endometrial carcinomas. In a recently presented phase II clinical trial (NCT03579316), the WEE1 inhibitor adavosertib showed promising clinical activity both as monotherapy and in combination with Olaparib, in patients with recurrent ovarian cancer irrespective of BRCA mutational status. Adavosertib has also been tested in combination with chemotherapy in platinum resistant settings with significant efficacy. The most promising combination was with carboplatin showing disease control in all treated patients, but the combination needs further optimization due to increased hematological toxicity [99]. Finally, in a phase II randomized trial, adavosertib combined with gemcitabine provided significant benefit in mPFS in comparison to gemcitabine alone in patients with platinum resistant recurrence of the disease [100], warranting further investigation in a larger confirmatory trial.

### 4.4. Immunotherapy

The role of immunotherapy is currently under investigation in advanced ovarian cancer. Based on molecular profile analysis, only a small percentage of ovarian carcinomas have increased tumor mutational burden (TMB) or T cell–inflamed gene expression profile (GEP) favoring response to immunotherapy [101]. Indeed, phase III clinical trials conducted so far with the addition of anti-PD1/anti-PD-L1 antibodies to standard treatment in either frontline setting or recurrent disease were negative [102,103]. Introduction of PARPi in the therapeutic algorithm of ovarian cancer has provided the rational for combinations with immune-checkpoint inhibitors since PARPi has been shown to activate the STING pathway [104]. Early phase clinical trials have provided clinical evidence of activity [105,106], and the results of three phase III randomized clinical trials evaluating the efficacy of PARPi-immunotherapy combinations as maintenance treatment after platinum doublet chemotherapy in the frontline setting of the disease are eagerly anticipated.

In addition, microsatellite instability has been recognized in approximately 3% of ovarian cancer patients [101]. Whole exome sequencing analysis has revealed an increased number of mutations in MSI-high patients related to exceptional clinical responses to immunotherapeutic agents [107]. Specifically, in recurrent ovarian cancer patients, the anti-PD-1 agent pembrolizumab has demonstrated 33% overall response rate in a phase II basket trial [108]. Despite there being no conclusive evidence regarding the role of mismatch repair genes status in acquired resistance to platinum agents, there are reports in the literature indicating that the expression of MSH2 and MLH1 may be altered due to platinum-based chemotherapy [109]. The latter deserves further investigation to examine potential activity of immunotherapeutic agent combinations in ovarian cancer patients previously treated with platinum.

## 5. Discussion

For the last thirty years, the combination of carboplatin and paclitaxel has been the standard of care for OC patients. Although more than 80% of these patients will initially have a response to therapy, the majority will ultimately experience disease recurrence and eventually develop chemotherapy-resistant disease. Most relapses will not be curable as treatment efficacy decreases with time. Quality of their life is severely impaired due to various manifestations of the disease, more commonly bowel obstruction, ascites, and pleural effusion. Therefore, new treatment strategies are of great need for these patients. Among these, immunotherapy has attracted significant interest due to recent improvements in the understanding of the molecular basis of immune recognition and immune regulation of cancer cells [110]. However, despite the high proportion of HRD ovarian cancers with suspected high tumor mutational burden (TMB), increased infiltration by CD8+ tumor-infiltrating lymphocytes (TILs), and high expression of tumor antigens capable of eliciting spontaneous anti-tumor responses, initial attempts using immunotherapy in ovarian cancer were largely disappointing [111].

Interestingly, due to the fundamental dependence of cancer cells upon DDR pathways, DNA repair-targeted agents such as platinum drugs represent an exciting group of emerging therapeutics. Thus, a better understanding of DDR alterations might have potential implications in OC treatment. Extensive research has been carried out on the relationship between DDR changes and response to platinum-based drugs, but results are contradictory. Methodological differences may account up to a point for these discrepancies. The complexity of molecular mechanisms underlying resistance to chemotherapy and the polyclonality of OC represent significant gaps in our knowledge. For example, although metastatic ovarian tumors mimic the primary ovarian neoplasm morphologically and clinically, protein analysis in biopsy of primary tumors has been shown to be different from that of metastases.

On the other hand, the success of PARP inhibitors in HR-deficient ovarian cancer highlights the potential of DDR modifiers. An increasing number of studies on DNA repair pathways including DNA repair gene expression profiling, mutation status of DNA repair genes, expression levels of DNA repair proteins, and DNA repair capacity have been demonstrated to have a predictive value for the response to therapies in different types of cancer. These data suggest that the assessment of the activity of DNA repair pathways in tumor cells may identify new therapeutic targets and novel biological markers that may influence clinical decision making. Several DDR inhibitors, including those targeting ATM, ATR, DNA-PK, Chk1, and Wee1 have already entered into clinical trials [112]. Predictive biomarkers, which have been extensively validated preclinically, can be utilized in DDR inhibitor clinical trial design to define the most suitable patient population. Furthermore, to ensure that clinical studies generate useful mechanistic observations, clinical trials of DDR inhibitors should incorporate pharmacodynamic biomarkers that can molecularly investigate whether a drug hits the desired target.

## 6. Conclusions

Taken together, the results reviewed herein suggest that deregulated DDR network plays a crucial role in the cisplatin resistance in ovarian cancer. Thus, unravelling these molecular pathways can be exploited to discover new treatment opportunities in the field. However, taking into account the complexity of the DDR, we need to fully understand the interplay between molecular factors that promote either death or survival of ovarian cancer cells. This new knowledge is essential to design future strategies to circumvent the complex mechanisms of cisplatin resistance more effectively and to translate them into improved clinical responses.
